# Serum zinc concentration and history of isCGM contact dermatitis in type 1 diabetes

**DOI:** 10.1186/s41043-025-00927-x

**Published:** 2025-05-17

**Authors:** Mitsunobu Kubota, Shizuka Matsuda, Mimu Matsuda, Shinji Maeda, Sayo Yoshiyama

**Affiliations:** Department of Endocrinology and Diabetology, National Hospital Organization Kure Medical Center and Chugoku Cancer Center, 3-1 Aoyamacho, Kure, Hiroshima, 737-0023 Japan

**Keywords:** Serum zinc concentration, Intermittent scanning continuous glucose monitor, Type 1 diabetes, Contact dermatitis

## Abstract

**Background:**

In insulin treatment for type 1 diabetes, intermittent scanning continuous glucose monitoring (isCGM: FreeStyle® Libre), in which a sensor is adhered to the skin, is often used to monitor blood glucose fluctuations and manage glucose levels. Zinc-deficient skin is reportedly more susceptible to primary irritant rashes. This study investigated whether zinc deficiency is associated with a history of contact dermatitis caused by isCGM in patients with type 1 diabetes.

**Methods:**

The subjects comprised 55 patients (23 men, 32 women, age 57.9 ± 17.6 years) with type 1 diabetes who were outpatients at our department and had a history of isCGM use. We examined the history of contact dermatitis due to isCGM in relation to serum zinc concentration.

**Results:**

Serum zinc was significantly lower in those with history of contact dermatitis (23 subjects) compared to those without (32 subjects) (*P* = 0.033). History of contact dermatitis due to isCGM was negatively associated with both age (β =  − 0.266, *P* = 0.040) and zinc deficiency category (β =  − 0.315, *P* = 0.017).

**Conclusions:**

For people undergoing treatment for type 1 diabetes for whom skin problems caused by isCGM are a barrier to glucose management, screening of serum zinc concentration may be important.

## Background

In recent years, it has been reported that diabetes patients tend to have lower zinc intake due to an unbalanced diet resulting from changes in lifestyle habits and increased rate of eating disorders [1]. Although few reports have indicated direct association of zinc deficiency with diabetes, a cohort study in the United States has shown that women with low zinc intake have increased risk of developing diabetes [2]. Moreover, zinc deficiency exacerbates insulin resistance in non-insulin-dependent diabetics [3], and serum zinc levels are both inversely correlated with HbA1c in type 2 diabetes [4] and associated with diabetic peripheral neuropathy [5]. It has been reported that even when zinc intake is about 10 mg/day, diabetic patients are likely to become zinc deficient [6]. The mechanisms of zinc deficiency include breakdown of fat and muscle due to diabetes progression (with consequent excretion of the zinc contained in those tissues), impaired zinc absorption in the gastrointestinal tract [7], and renal damage due to diabetic nephropathy, which increases urinary zinc excretion; additionally, if proteinuria increases in diabetic kidney disease, zinc loss also increases [8, 9]. In fact, we reported identification of zinc deficiency or subclinical zinc deficiency in 57% of subjects with poor glycemic control, a relationship of serum zinc concentration to the severity of chronic kidney disease associated with diabetes, and potential relation of zinc deficiency to sarcopenia associated with diabetes [10]. Zinc deficiency can cause various disorders in diabetic patients, such as anemia, delayed wound healing, decreased reproductive function, decreased sense of taste, dermatitis, stomatitis, and hair loss [11]; accordingly, it is considered necessary to pay attention to serum zinc concentration in diabetes therapy.

**Fig. 1:**
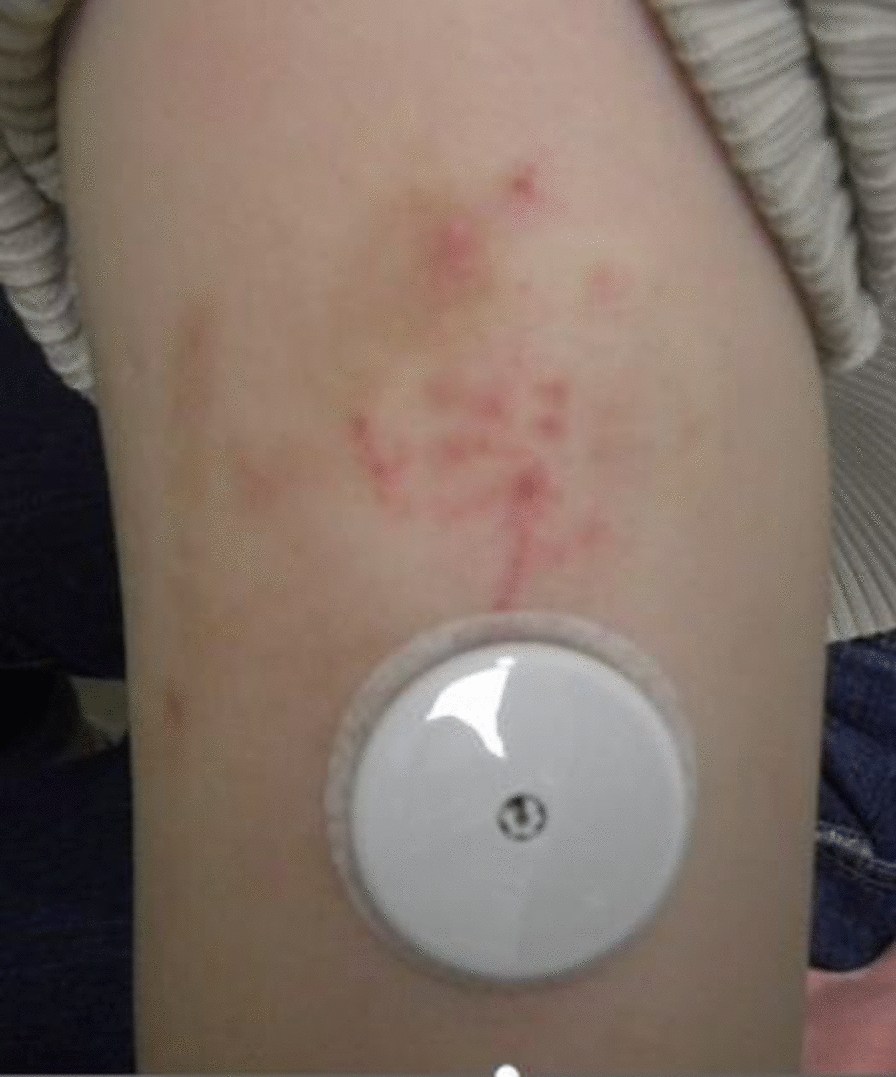
Representative image of isCGM on the right upper arm of a person with type 1 diabetes. Photograph of contact dermatitis caused by isCGM on the right upper arm of a woman in her 20 s with type 1 diabetes, who was one of the participants in this study

The use of an intermittent scanning continuous glucose monitor (isCGM: FreeStyle® Libre, Abbott Japan LLC, Tokyo, Japan) for blood glucose management is becoming more common in Japan. Particularly in the treatment of type 1 diabetes, blood glucose fluctuations are large due to severe reduction in endogenous insulin secretion, and managing the disease necessitates treatment with frequent insulin injections or continuous subcutaneous insulin infusion. Hence, there is a high need to use isCGM to check blood glucose fluctuations and adjust the amount of insulin injected accordingly. In the course of such therapy, some patients complain of contact dermatitis caused by isCGM. A typical image of such dermatitis is shown in Fig. 1. In severe cases, the use of isCGM is discontinued due to itching and exudate, as either can cause sensor peeling and make blood glucose management more difficult. Notably, in epithelial tissues, the zinc transporter ZIP10 regulates intracellular zinc and optimizes the transcriptional activity of p63, a transcription factor important in epidermal development, thereby contributing to the formation of epithelial tissue and maintenance of skin barrier function [12]. It has been reported that in zinc-deficient skin, irritants cause excess ATP to be released from epidermal cells, promoting infiltration of neutrophils and the release of chemokines, which are inflammatory cytokines; additionally, decreased abundance of Langerhans cells causes a prolonged skin inflammatory response, making primary irritant rashes more likely to occur [13]. The purpose of this study was to investigate whether zinc deficiency is associated with a history of contact dermatitis caused by isCGM in type 1 diabetes.

## Methods

### Study group

We enrolled 55 subjects (23 men, 32 women, age 57.9 ± 17.6 years) with type 1 diabetes who were outpatients at our department and had a history of isCGM use (FreeStyle® Libre, Abbott Japan LLC, Tokyo, Japan) for diabetes treatment during the period from April 2023 to March 2024. All subjects with type 1 diabetes were treated with frequent self-injections of insulin or with continuous subcutaneous insulin infusion. For each patient, we recorded duration of diabetes, body weight, glucose metabolism index, and serum zinc concentration. Additionally, we identified and recorded whether they had a history of contact dermatitis due to isCGM. Each participant was interviewed and provided informed consent for publication of their data or clinical images. This study was approved by the Ethics Committee of the National Hospital Organization of Kure Medical Center (file number 30–41).

### Biochemical analyses

After overnight fasting, each participant underwent a physical examination and venous blood collection early in the morning. Body measurements were taken in the standing position. Collected blood and urine samples were centrifuged and measured by appropriate method. Plasma glucose levels were measured by the glucose oxidase method and HbA1c levels by high performance liquid chromatography (HPLC) on a HLC723-G9 (Tosoh, Tokyo, Japan). Serum zinc concentration was measured using the ACCURAS AUTO Zn reagent kit (Shino-Test Corporation, Japan; LOD, 1.87 μg/dL; LOQ-CV10%, 5.312 μg/dL; range of measurement, 4.0–500 μg/dL; CV, 1.08%), which can be employed with all auto-analyzers widely used in hospital laboratories and does not need any serum pretreatment [14]. Serum zinc levels were categorized according to the criteria of the Japanese Society of Clinical Nutrition [15]: normal, ≥ 80 μg/dL; subclinical zinc deficiency, ≥ 60 μg/dL and < 80 μg/dL; and zinc deficiency, < 60 μg/dL.

### Statistical analysis

Data are expressed as mean ± S.D. or median (25 th–75 th percentiles) depending on the data distribution. Due to having skewed distributions, TG, eGFR, and UACR values were logarithmically transformed before analysis. Differences in continuous variables between subcategories were tested for significance using analysis of covariance. Categorized variables were analyzed using the *χ*^2^ test. Spearman’s correlation coefficient (r) and *P*-values were determined for univariate correlation of serum Zn with metabolic variables. For analysis purposes, history of contact dermatitis due to isCGM was coded as 0 for no history and 1 for history. Similarly, zinc deficiency was categorically coded as 0 for deficiency, 1 for subclinical deficiency, and 2 for normal level. *P-*values < 0.05 were considered statistically significant. All analyses were performed using the software package SPSS version 29 (IBM Co. Ltd., Armonk, NY, USA).

## Results

**Fig. 2:**
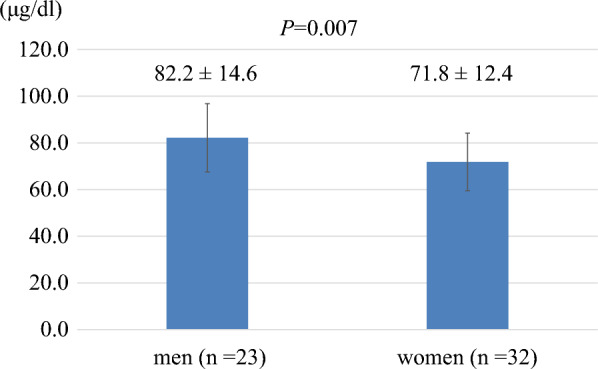
Serum zinc concentration in type 1 diabetes by gender. Serum zinc concentrations were 82.2 ± 14.6 μg/dL in men (n = 23) and 71.8 ± 12.4 μg/dL in women (n = 32). Women had significantly lower serum zinc than men (*P* = 0.007). Significance was determined by analysis of covariance. Columns and error bars indicate mean ± standard deviation (S.D.)

The subjects consisted of 55 patients with type 1 diabetes (23 men, 32 women, age 57.9 ± 17.6 years, age at onset of diabetes 41.0 ± 18.9 years, duration of diabetes 18.3 ± 14.5 years, BMI 23.2 ± 3.9, HbA1c 7.8 ± 2.1%, serum C-peptide 0.3 ± 0.5 ng/dL). The mean serum zinc concentration was 76.2 ± 14.2 μg/dL, and zinc level was categorized as normal in 22 patients, subclinical zinc deficiency in 27 patients, and zinc deficiency in 6 patients. Twenty-three patients had a history of contact dermatitis due to isCGM, while 32 patients had no such history (Table 1). Average serum zinc concentrations were 82.2 ± 14.6 μg/dL in men and 71.8 ± 12.4 μg/dL in women, with women on balance exhibiting significantly lower concentration than men (Fig. 2). Serum zinc concentration was positively correlated with age (r = 0.273, *P* = 0.044), age at onset of diabetes (r = 0.316, *P* = 0.019), and serum albumin concentration (r = 0.294, *P* = 0.029). Notably, serum zinc concentration showed a significant correlation with binary history of isCGM contact dermatitis (r =  − 0.288, *P* = 0.033). There was no significant association of serum zinc with either HbA1c, an index of blood glucose control, or duration of diabetes (Table 2).

**Table 1:** Baseline characteristics of participants

N (Men/Women)	55 (23/32)
Age, *years*	57.9 ± 17.6
Age at onset of diabetes, *years*	41.0 ± 18.9
Duration of diabetes, *years*	18.3 ± 14.5
Height, cm	160.1 ± 9.3
Body weight, kg	59.5 ± 11.1
BMI, kg/m^2^	23.2 ± 3.9
HbA1c, %	7.8 ± 2.1
Fasting serum C-peptide, ng/dL	0.31 ± 0.52
TC, mg/dl	191.2 ± 40.6
LDL-C, mg/dl	98.9 ± 35.6
TG, mg/dl	90.0 (66.0–131.0)
HDL-C, mg/dl	66.4 ± 20.0
TP, mg/dl	6.8 ± 0.8
Alb, mg/dl	4.0 ± 0.6
Estimated GFR, ml/min/1.73 m^2^	79.7 (65.9–96.1)
Urinary albumin-to-creatinine ratio, mg Alb/g Cre	11.0 (7.0–31.0)
Diabetic nephropathy stage (1/2/3/4/5)	41/10/2/2/0
Diabetic retinopathy stage	
(NDR/SDR/PPDR/PDR)	43/5/2/5
Zn, μg/dL	76.2 ± 14.2
Serum zinc classification	
(Normal/Subclinical deficiency/Deficiency)	22/27/6
History of contact dermatitis due to isCGM (Yes/No)	23/32

BMI, body mass index; HbA1c, hemoglobin A1c; TC, total cholesterol; LDL, low-density lipoprotein; HDL, high-density lipoprotein; TG, triglyceride; TP, total protein; Alb, albumin; GFR, glomerular filtration rate; NDR, no diabetic retinopathy; SDR, simple diabetic retinopathy; PPDR, pre-proliferative diabetic retinopathy; PDR, proliferative diabetic retinopathy; serum zinc classification: normal, ≥ 80 μg/dL; subclinical deficiency, ≥ 60 and < 80 μg/dL, deficiency, < 60 μg/dL. isCGM, intermittent scanning continuous glucose monitoring (FreeStyle® Libre)

Data are presented as number, mean ± S.D., or median (25 th‒75 th percentiles)

**Table 2:** Relationship between serum zinc concentration and metabolic parameters in all subjects

Serum zinc concentration		
	*r*	*P*
Age, *years*	0.273	0.044
Sex,* men/women*	− 0.364	0.006
Age at onset of diabetes, *years*	0.316	0.019
Duration of diabetes, *years*	− 0.070	0.613
Body weight, kg	0.092	0.503
BMI, kg/m^2^	0.005	0.972
HbA1c, %	− 0.085	0.538
Fasting serum C-peptide, ng/dL	0.106	0.442
TC, mg/dl	0.141	0.304
LDL-C, mg/dl	0.255	0.060
TG, mg/dl	0.076	0.582
HDL-C, mg/dl	− 0.146	0.286
TP, mg/dl	0.157	0.284
Alb, mg/dl	0.294	0.029
Estimated GFR, ml/min/1.73 m^2^	− 0.047	0.734
Urinary albumin-to-creatinine ratio, mg Alb/g Cre	− 0.046	0.739
History of contact dermatitis due to isCGM (Yes/No)	− 0.288	0.033

Univariate correlations of serum zinc concentration with measured variables in all subjects. Spearman’s rank correlation coefficients (r) and P-values are presented. BMI, body mass index; HbA1c, hemoglobin A1c; TC, total cholesterol; LDL, low-density lipoprotein; HDL, high-density lipoprotein; TG, triglyceride; TP, total protein; Alb, albumin; GFR, glomerular filtration rate; isCGM, intermittent scanning continuous glucose monitoring (FreeStyle® Libre)

**Fig. 3:**
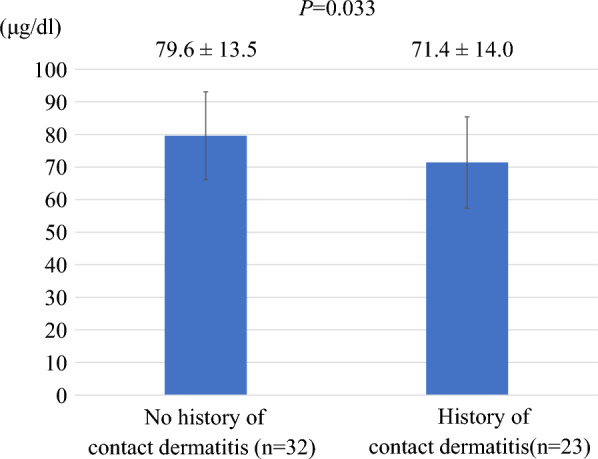
Serum zinc concentration according to the presence or absence of a history of contact dermatitis due to isCGM. Serum zinc concentrations were 79.6 ± 13.5 μg/dL in 32 subjects with no history of dermatitis due to isCGM and 71.4 ± 14.0 μg/dL in 23 subjects with such history, with the latter being significantly lower (*P* = 0.033). Significance was determined by analysis of covariance. Columns and error bars indicate mean ± standard deviation (S.D.)

The serum zinc concentration was 79.6 ± 13.5 μg/dL in subjects with no history of contact dermatitis by isCGM (n = 32) and 71.4 ± 14.0 μg/dL in subjects with such history (n = 23), the latter being significantly lower (*P* = 0.033) (Fig. 3). When subjects were classified according to degree of zinc deficiency, the number per group with history of contact dermatitis was 7 in the normal group (n = 22), 10 in the subclinical zinc deficiency group (n = 27), and 6 in the zinc deficiency group (n = 6). Application of the *χ*^2^ test to this cross-tabulation detected a bias, in which subjects with history of contact dermatitis were overrepresented among the zinc-deficient and subclinical zinc-deficient groups (*P* = 0.009) (Table 3).

**Table 3:** Proportion of patients with isCGM-related contact dermatitis by categorical serum zinc level

	No history of isCGM contact dermatitis	History of isCGM contact dermatitis	Total *P* = 0.009
Normal	15	7	22
Subclinical deficiency	17	10	27
Deficiency	0	6	6
Total	32	23	55

Serum zinc levels were categorized according to the criteria of the Japanese Society of Clinical Nutrition [15]: normal, ≥ 80 μg/dL; subclinical zinc deficiency, ≥ 60 μg/dL and < 80 μg/dL; and zinc deficiency, < 60 μg/dL. Categorized variables were analyzed using the χ^2^ test and the *P* value shown is for the category analysis

Finally, to clarify the relationship between history of contact dermatitis due to isCGM and zinc deficiency, we incorporated the potential confounders identified as having significant relation with serum zinc concentration in Table 2 into the multiple regression model (Table 4). This revealed history of contact dermatitis due to isCGM to be negatively associated with both age (β =  − 0.266, *P* = 0.040) and zinc deficiency category (β =  − 0.315, *P* = 0.017).

**Table 4:** Multiple regression analysis of the relationship between history of contact dermatitis due to isCGM and parameters related to serum zinc concentration in the study subjects

History of contact dermatitis due to isCGM		
	*β*	*P*
Age, *years*	− 0.266	0.040
Sex,* men/women*	0.172	0.169
Alb, mg/dl	0.047	0.700
Zinc deficiency category	− 0.315	0.017

β, standardized regression coefficient. A multiple regression analysis was performed with history of contact dermatitis due to isCGM as the dependent variable and parameters found to correlate with serum zinc concentration as explanatory variables. R^2^ = 0.279, *P* = 0.002. Serum zinc levels were categorized according to the criteria of the Japanese Society of Clinical Nutrition

## Discussion

This study is the first to show that serum zinc concentrations are low in type 1 diabetes patients with a history of contact dermatitis due to isCGM. In addition, zinc deficiency was found to be independently associated with history of contact dermatitis in type 1 diabetes. This suggests that screening for zinc deficiency is important in cases of type 1 diabetes mellitus for which isCGM is necessary but contraindicated due to contact dermatitis.

Among the subjects in this study, women had lower serum zinc concentrations than men (Fig. 2), and there was a tendency for serum zinc concentration to be lower in those with younger age and younger age at onset of diabetes (Table 2). It has been reported that patients with diabetes tend to have lower zinc intake due to an unbalanced diet resulting from changes in lifestyle habits and increased rate of eating disorders, especially among women [1]. Zinc is abundant in oysters, beef, liver, seafood, cereals, beans, nuts, and cheese [1], and is important as a cofactor for many enzymes, including the antioxidant enzymes catalase, peroxidase, and superoxide dismutase [16]. In type 1 diabetes treatment, as isCGM becomes more widespread, it becomes easier for people to recognize fluctuations in their blood glucose levels, which raises concerns that out of fear of their blood glucose rising, they sometimes avoid high-calorie foods and may not consume enough foods containing high-quality protein. These choices may result in reduced intake of zinc.

Under hyperglycemia, production of free radicals such as superoxide is enhanced, resulting in microangiopathies such as diabetic nephropathy and atherosclerosis [17]. Superoxide dismutase (SOD), which uses Cu and Zn as cofactors, is an important protective agent and inactivator of superoxide [18]; however, its activity in microvascular walls is decreased by glycation due to hyperglycemia [19] and by zinc deficiency [20]. As a result, production of superoxide in the vascular wall tends to increase, resulting in increased formation of peroxynitrite, which in turn leads to a lack of action of the vasodilator NO. This mechanism may be associated with skin microcirculation in diabetes in the context of zinc deficiency [21]. In addition, it has been reported that low serum zinc concentration can worsen skin pruritus and impair skin barrier function [22]. Furthermore, in zinc-deficient skin, irritants cause epidermal cells to release excess ATP, which promotes infiltration of neutrophils and the release of chemokines, which are inflammatory cytokines. In addition, a decrease in epidermal Langerhans cells (LCs), which promote suppression of inflammatory responses, has been reported to cause prolongation of the skin inflammatory response, making the skin more susceptible to primary irritant rashes [13]. In a study using skin tissue from dermatitis patients, an initially significant depletion of LCs was alleviated by zinc supplementation, occurring alongside recovery from dermatitis [23].

In a report by Pyl et al., patch tests identified 3.8% of isCGM users as having allergic contact dermatitis to the allergen isobornyl acrylate, and the protective film was determined not very effective [24]. In this study, 42% of patients had a history of contact dermatitis due to isCGM. Notably, we counted subjects as having history of contact dermatitis if they experienced any dermatitis at least once, without requirement of a patch test. Hence, in addition to allergic contact dermatitis, we might have included irritant contact dermatitis, immediate skin reaction, photoinduced contact dermatitis, systemic contact dermatitis, and non-eczematous contact dermatitis, which likely explains the difference in the observed frequency of contact dermatitis in isCGM. Furthermore, we only included patients with type 1 diabetes, among which population contact dermatitis may be more frequent due to autoimmune mechanisms. Most physicians who treat type 1 diabetes have likely encountered a case of contact dermatitis due to isCGM and faced difficulties in treating it. In 2019, a study in Japan identified isobornyl acrylate contained in the tape as the causative allergen in contact dermatitis caused by isCGM with the Freestyle® Libre [25]. The next year, the product offered in Japan was improved to one free of isobornyl acrylate, and although details have not been released officially, it seems that the frequency of contact dermatitis due to isCGM may have decreased in actual clinical practice. Nevertheless, contact dermatitis due to isCGM still sometimes occurs in the treatment of type 1 diabetes, and in severe cases, itching and sensor peeling caused by exudate may necessitate abandoning the use of isCGM, making blood glucose management more difficult.

### Study limitations

There are some limitations of this research. First, this was a retrospective observational study with a limited number of patients. Prospective studies remain needed to determine whether zinc intake can improve contact dermatitis due to isCGM. Second, this study was limited to Japanese subjects with type 1 diabetes. It is not certain that similar results would be obtained in subjects with type 2 diabetes or in people of other races. Third, data on the history of contact dermatitis due to isCGM were compiled based on entries in a database of medical records in which physicians evaluated and recorded the presence or absence of contact dermatitis due to isCGM when examining patients with type 1 diabetes. We were not able to diagnose the specific type of contact dermatitis or characterize allergen sensitivities through assays such as patch tests, skin tests, or serum allergen-specific IgE tests for definitive diagnosis.

## Conclusions

This study suggests that in people with type 1 diabetes for whom skin problems caused by isCGM are a barrier to treatment, screening serum zinc concentration may be helpful. Future prospective trials are anticipated to demonstrate that zinc supplementation in zinc-deficient individuals can prevent contact dermatitis.

## Data Availability

Availability of data and material the datasets used/or analysed during the current study available from the corresponding author on reasonable request.

## Abbreviations

eGFR: Estimated glomerular filtration rate
UACR: Urinary albumin-to-creatinine ratio
BMI: Body mass index
SMI: Skeletal muscle mass index
TC: Total cholesterol
TG: Triglyceride
HDL-C: High-density lipoprotein cholesterol
LDL-C: Low-density lipoporotein cholesterol
HbA1c: Hemoglobin A1c
TP: Total protein
Alb: Albumin
isCGM: Intermittent scanning continuous glucose monitoring (FreeStyle® Libre)
